# Real-World Clinical Outcomes of Azacitidine Versus Best Supportive Care in Higher-Risk Myelodysplastic Neoplasms: A Single-Center Cohort Study

**DOI:** 10.3390/medsci14020212

**Published:** 2026-04-24

**Authors:** Mihai-Emilian Lapadat, Oana Stanca, Irina Nicoleta Triantafyllidis, Anca Mariana Ciobanu, Nicoleta Mariana Berbec, Cristina Negotei, Cristian Tudor Barta, Ana Maria Bordea, Madalina Marilena Oprea, Andrei Colita

**Affiliations:** 1 Department of Hematology, University of Medicine and Pharmacy Carol Davila, 050474 Bucharest, Romania; mihai-emilian.lapadat@umfcd.ro (M.-E.L.); irina.triant@umfcd.ro (I.N.T.); nicoleta.berbec@umfcd.ro (N.M.B.); cristian.barta@umfcd.ro (C.T.B.); andrei.colita@umfcd.ro (A.C.); 2 Clinic of Hematology, Coltea Clinical Hospital, Bld IC Bratianu 1-3, 030171 Bucharest, Romania; ancaciobanu05@yahoo.com (A.M.C.); cristina.negotei@gmail.com (C.N.); ana_maria_ivanescu@yahoo.co.uk (A.M.B.); mady_1969@yahoo.com (M.M.O.)

**Keywords:** higher-risk myelodysplastic neoplasms, azacitidine, hypomethylating agents, overall survival, leukemic transformation, IPSS-R, real-world cohort

## Abstract

**Background**: Higher-risk myelodysplastic neoplasms (HR-MDS) are associated with poor survival and a substantial risk of leukemic transformation. Although azacitidine is a standard treatment in this setting, comparative real-world data remain limited. We evaluated the association between azacitidine exposure and clinical outcomes in patients with HR-MDS. **Methods**: We performed a retrospective single-center cohort study including 72 adults with HR-MDS, defined by the Revised International Prognostic Scoring System(IPSS-R) as High or Very High risk. Patients were categorized as azacitidine-treated (Aza, *n* = 44) or managed with best supportive care alone (No Aza, *n* = 28). Overall survival (OS) was defined from diagnosis to death from any cause. Progression-free survival (PFS) was defined as the time to acute myeloid leukemia transformation or death. Leukemic transformation (LT) was analyzed using Kaplan–Meier estimates and competing-risk cumulative incidence, with death without prior LT treated as a competing event. Univariable and multivariable Cox regression models were applied. **Results**: Azacitidine exposure was associated with longer OS compared with best supportive care, with a median OS of 13 vs.10 months and 24-month OS rates of 27% vs. 14% (*p* = 0.0239). PFS was also prolonged in the Aza group, with a median of 11 vs. 7 months (*p* = 0.0406). LT-related outcomes similarly favored azacitidine. In multivariable analyses, azacitidine remained independently associated with improved OS, PFS, and LT-related outcomes. IPSS-R Very High risk remained an adverse prognostic factor across endpoints, while a higher baseline bone marrow blast percentage independently predicted leukemic transformation. **Conclusions**: In this real-world HR-MDS cohort, azacitidine exposure was associated with improved survival outcomes and delayed leukemic transformation compared with best supportive care alone.

## 1. Introduction

Myelodysplastic neoplasms, formerly known as myelodysplastic syndromes (MDSs), are clonal hematopoietic stem cell diseases characterized by marked biological and clinical heterogeneity with variable acute myeloid leukemia (AML) transformation risk [1]. Disease trajectories differ according to baseline characteristics, including the degree of marrow dysplasia, affected lineages, and—most critically—cytogenetic abnormalities present at diagnosis that can drive clonal evolution and leukemic transformation [2].

Given this heterogeneity, early and accurate risk stratification is essential. Several prognostic tools—most commonly IPSS [3], IPSS-R [4], and WPSS [5], and more recently IPSS-M [6]—have been developed to standardize assessment. These scores incorporate clinical and cytogenomical variables to classify patients into two broader clinical categories—lower-risk and higher-risk MDS—with differing expectations for survival and risk of AML evolution.

The necessity for accurate risk stratification is especially clear in therapeutic decision-making [7]. For lower-risk MDS, management emphasizes supportive strategies—red cell transfusions, erythropoiesis-stimulating agents (ESAs) [7], Luspatercept [8], antimicrobial prophylaxis, and iron chelation—tailored to symptom control and correction of cytopenias [9]. By contrast, higher-risk MDS generally warrants disease-modifying interventions. Hypomethylating agents (HMAs), particularly azacitidine [10], play a central role in routine practice, while allogeneic hematopoietic stem cell transplantation remains the only potentially curative option for select fit, younger patients [11].

Azacitidine is a hypomethylating agent that induces passive DNA hypomethylation across cell divisions and re-expression of silenced genes [12,13], with additional cytotoxic effects at higher exposures [12]. In the pivotal AZA-001 trial, azacitidine improved overall survival (OS) compared with conventional care regimens (24 vs. 15 months) and established itself as a standard of care for higher-risk disease [14].

Outcomes outside clinical trials, however, remain heterogeneous. Real-world cohorts commonly report shorter median survival than AZA-001 (6.1 to 15.0 months when stratified by adverse prognostic factors [15], around 13.4 months [16], 14.9 months [17], 16.5 months [18], up to 17.6 months [19], and 20 months in selected series [20]), reflecting differences in case mix, baseline frailty, and treatment delivery, including early discontinuation, dose reductions, and fewer cycles. A frequent limitation of observational series is the absence of a directly comparable untreated group within the same clinical context; many studies are all-azacitidine cohorts or compare against heterogeneous “conventional therapy” rather than explicit azacitidine versus no-azacitidine comparisons in one practice environment.

Evidence regarding progression-free survival (PFS) and leukemic transformation (LT) is even less consistent than for overall survival. PFS definitions vary across studies, and LT is often reported as crude proportions or with Kaplan–Meier methods that censor deaths, even though death frequently precludes observation of AML transformation in higher-risk MDS [21,22]. In the CALGB 9221 randomized trial, azacitidine prolonged PFS and time to leukemic transformation or death compared with supportive care (21 vs. 13 months), and reduced AML transformation as the first event (15% vs. 38%) [23]. Other cohorts have reported median PFS around 15 months with azacitidine [19], whereas randomized-trial meta-analysis showed improved time to transformation/death (pooled HR 0.69, 95% CI 0.58–0.82) [24]. Real-world first-line HMA cohorts have reported AML transformation rates of 27.8% at 1 year and 39.7% at 2 years among azacitidine/decitabine starters [25]. Together, these data indicate that endpoint definitions and handling of competing mortality can materially influence effect estimates. Concurrent reporting of overall survival, clinically anchored PFS (LT or death), and LT with competing-risk methods may therefore improve interpretability in real-world comparisons.

To address these issues, we examined a single-center, real-world cohort restricted to IPSS-R High and Very High categories and explicitly separated patients by treatment exposure into azacitidine-treated (Aza) and no-azacitidine/best supportive care (No Aza) groups. Within one care environment, we analyzed overall survival, progression-free survival, and leukemic transformation as distinct, prespecified endpoints and evaluated LT using both Kaplan–Meier methods and competing-risk cumulative incidence. We also explored whether prognostic heterogeneity persists within the higher-risk spectrum and which baseline factors independently predict outcomes.

By comparing treated and untreated patients within one center and one timeframe, we reduce cross-center variability in diagnostic work-up, follow-up intensity, and supportive care practice that can confound broader real-world comparisons, while acknowledging that treatment allocation in routine care remains non-random.

## 2. Materials and Methods

This was a retrospective cohort study of adult patients with confirmed MDS treated in a tertiary center over a five-year period. Diagnosis was established by bone marrow biopsy and cytogenetics. IPSS-R was calculated for all patients, and only higher-risk disease (IPSS-R High or Very High) was included. Data were abstracted from the institutional electronic medical record and a dedicated clinical database.

Patients were categorized into two groups based on treatment exposure: Aza (received azacitidine) and No Aza (received best supportive care). The No Aza/BSC group received best supportive care according to institutional practice. This included supportive interventions such as red blood cell and platelet transfusions, antimicrobial treatment/prophylaxis, erythropoiesis-stimulating agents in selected patients without excess blasts, and iron chelation therapy in chronically transfused patients where clinically indicated. These patients did not receive disease-modifying therapy with hypomethylating agents, low-dose cytarabine, or intensive chemotherapy. Treatment duration and number of cycles were recorded but were not used as covariates in survival models to avoid immortal time bias.

Primary outcomes were overall survival and progression-free survival; secondary outcomes included leukemic transformation and identification of independent predictors for OS, PFS and LT. OS was defined as time from baseline to death from any cause. PFS was defined as the earliest of LT or death (composite endpoint); patients without either event were censored at last contact. LT was defined as progression to AML according to accepted thresholds. LT was evaluated using (i) Kaplan–Meier transformation-free survival (deaths censored) and (ii) competing-risk cumulative incidence (Aalen–Johansen), treating death without prior LT as a competing event.

Baseline covariates considered for adjustment included demographics, IPSS-R category and cytogenetic risk group, transfusion dependence, marrow blast percentage and selected laboratory parameters. Multivariable models used complete-case analyses; covariates were considered for adjustment when at least 10 non-missing observations were available.

Time-to-event functions were estimated using Kaplan–Meier methods and compared between groups using the log-rank test. Cox proportional hazards regression estimated hazard ratios (HRs) with 95% confidence intervals (CIs). We first fit unadjusted Cox models for treatment (Aza vs. No Aza), then multivariable models adjusting for pre-specified baseline covariates.

To identify independent predictors for each endpoint (OS, PFS, LT), we implemented a purposeful selection strategy: variables with univariable *p* < 0.25 and clinically essential covariates (treatment, age, IPSS-R, karyotype) entered the initial model, backward elimination retained variables with *p* ≤ 0.10, and any variable whose removal changed the Aza HR by ≥10% was retained to control confounding, regardless of statistical significance. Model discrimination was summarized using Harrell’s concordance index (C-index).

Statistical analyses were performed using Python version 3.13.7 (Python Software Foundation, Wilmington, DE, USA). Data processing and database management were performed using pandas version 2.3.2 and NumPy version 2.3.3. Survival analyses, including Kaplan–Meier estimation, log-rank testing, Cox proportional hazards regression, Harrell’s concordance index, and competing-risk cumulative incidence using the Aalen–Johansen estimator, were performed using lifelines version 0.30.0. Graphical representations were generated using Matplotlib version 3.10.6.

## 3. Results

A total of 72 patients with IPSS-R High/Very High MDS were included. Forty-four received azacitidine (Aza) and 28 received best supportive care alone (No Aza). Median age at diagnosis was 71 years (range 31–89), and 40 patients (55.6%) were male. The median hemoglobin level at diagnosis was 7.2 g/dL (IQR 6.5–8.45), with 59/72 (82.0%) being transfusion-dependent. Neutropenia (ANC < 0.8 × 10^9^/L per IPSS-R criteria) was present in 22 patients (30.5%), and severe thrombocytopenia (<50 × 10^9^/L) in 18 (25.0%). The median bone marrow blast percentage was 11% (IQR 6–16; range 3–19), and most patients had excess blasts; these cases mapped to Refractory Anemia with Excess Blasts type 1 (22/72; 30.5%) and type 2 (38/72; 52.8%) using 2016 WHO categories, with the remainder largely classified as Refractory Citopenia with Multilineage Dysplasia (11/72; 15.2%).

According to IPSS-R cytogenetic risk categories, distributions were very good (2.8%), good (37.5%), intermediate (40.3%), poor (6.9%), and very poor (12.5%), with intermediate most frequent. A normal karyotype (good) was observed in 26/72 (36.1%). Complex karyotype (12.5%), trisomy 8 (9.7%), and chromosome 3 abnormalities (5.6%) were among the more common abnormalities. Baseline characteristics by treatment group are summarized in Table 1.

Median potential follow-up estimated by reverse Kaplan–Meier method was 56 months overall (95% CI 38–63 months by bootstrap); it was 56 months in the Aza group, and not estimable in the No Aza group due to few long-term censored observations. During follow-up, 59/72 (81.9%) patients died, 60/72 (83.3%) experienced a PFS event (LT or death), and 45/72 (62.5%) transformed to AML. Event proportions were similar between groups (death: 79.5% Aza vs. 85.7% No Aza; LT: 59.1% vs. 67.9%), but time-to-event analyses consistently favored azacitidine.

Accounting for time at risk, OS person-time totaled 69.5 person-years in the Aza group and 26.3 person-years in the No Aza group; for LT analyses, person-time was 56.1 and 19.7 person-years, respectively. Crude event rates per 100 person-years favored azacitidine: deaths 50.4 vs. 91.1; rate ratio (RR) 0.55 (95% CI, 0.33–0.93); LT events 46.4 vs. 96.6; RR 0.48 (95% CI, 0.27–0.87). These rate-based estimates aligned with Cox HRs.

Exposure to treatment was heterogeneous: 22/44 (50%) received ≥ 6 cycles and 13/44 (29.5%) received ≥ 12 cycles; maximum exposure was 60 months. Median treatment duration was 6 months (IQR, 3.0–12.25; range, 1–60); accordingly, response was assessed only at the 6-month time point. Best overall response comprised progressive disease (PD) with LT in eight patients (18.2%), partial (PR) or complete response (CR) in 9/44 (20.5%), and stable disease (SD) in 27/44 (61.4%).

**Figure 1:**
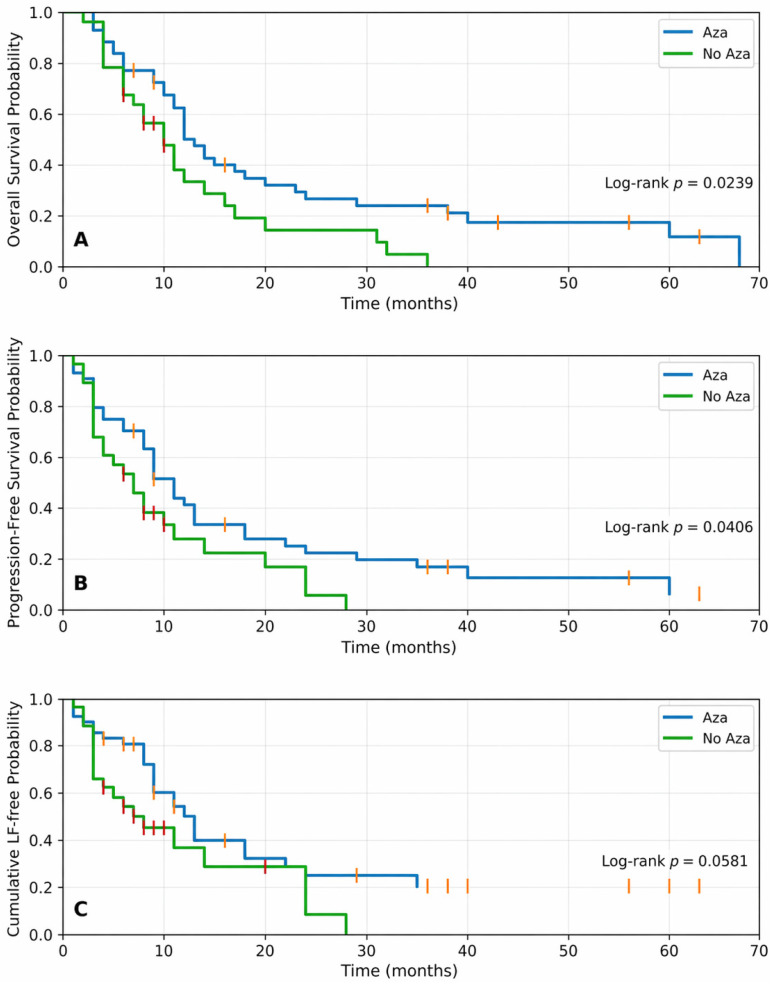
Kaplan–Meier estimates by azacitidine exposure (Aza vs. No Aza): (**A**) overall survival (OS); (**B**) progression-free survival (PFS); (**C**) leukemia transformation-free survival (LTFS). Vertical tick marks indicate censored observations and are shown in orange/red for visual distinction.

Regarding overall survival, the median OS for the entire cohort was 12 months. In subgroup analyses, the median OS was 13 months in the Aza group (35 deaths) and 10 months in the No Aza group (24 deaths). Estimated survival at 12 and 24 months was 50% and 27% with Aza versus 33% and 14% with No Aza (log-rank *p* = 0.0239) (Figure 1). Medians and milestone survival estimates at 12 and 24 months for OS, PFS and LT are shown in Table 2. In unadjusted Cox regression, azacitidine exposure was associated with a lower hazard of death (HR 0.55, 95% CI 0.32–0.95; *p* = 0.031). In the multivariable Cox model adjusting for baseline covariates, the association strengthened (HR 0.39, 95% CI 0.22–0.72; *p* = 0.003).

Median PFS for the entire cohort was 9 months. PFS was longer in the Aza group, with a median of 11 months (36 events) vs. 7 months in the No Aza group (24 events). Estimated PFS at 12 and 24 months was 41% and 22% with Aza vs. 28% and 6% with No Aza (log-rank *p* = 0.0406) (Figure 1). In unadjusted Cox regression, azacitidine showed a borderline association with improved PFS (HR 0.59, 95% CI 0.34–1.01; *p* = 0.053). After multivariable adjustment, azacitidine was independently associated with longer PFS (HR 0.39, 95% CI 0.20–0.73; *p* = 0.003).

In Kaplan–Meier analysis censoring deaths, median transformation-free survival was 13 months in the Aza group (26 events) vs. 8 months in the No Aza group (19 events), with a borderline between-group difference (log-rank *p* = 0.0581) (Figure 1). Estimated transformation-free survival at 12 and 24 months was 53% and 29% with Aza vs. 40% and 11% with No Aza. Treating death without prior transformation as a competing event (Aalen–Johansen estimator), the cumulative incidence of LT at 12 and 24 months was 42.4% and 61.3% with Aza vs. 61.7% and 72.8% with No Aza. In cause-specific Cox regression (deaths censored), azacitidine was associated with a lower hazard of transformation in univariable analysis (HR 0.57, 95% CI 0.31–1.05; *p* = 0.071), and the association strengthened after multivariable adjustment (HR 0.30, 95% CI 0.14–0.63; *p* = 0.001). Treatment effect estimates (Aza vs. No Aza) from unadjusted and adjusted Cox models for OS, PFS, and LT are shown in Table 3.

In purposeful-selection multivariable Cox models, IPSS-R risk group remained a dominant predictor across endpoints despite restricting inclusion to higher-risk disease. For OS, IPSS-R Very High versus High was associated with worse survival (HR 2.68, 95% CI 1.40–5.12; *p* = 0.003), while azacitidine remained independently associated with improved OS (HR 0.48, 95% CI 0.25–0.90; *p* = 0.023); albumin was retained for confounding control but was not statistically significant. For PFS, IPSS-R risk group (HR 2.61, 95% CI 1.42–4.80; *p* = 0.002) and azacitidine exposure (HR 0.34, 95% CI 0.18–0.63; *p* = 0.001) were independent predictors; marrow blasts were retained because they influenced the treatment estimate but did not reach statistical significance. For LT, IPSS-R risk group (HR 3.46, 95% CI 1.67–7.16; *p* = 0.001), azacitidine exposure (HR 0.23, 95% CI 0.10–0.48; *p* = 0.0001), and marrow blast percentage (HR 1.08 per 1% increase, 95% CI 1.01–1.16; *p* = 0.026) were independent predictors; age was retained because it influenced the treatment estimate. Final purposeful-selection Cox models (including covariates retained for confounding control) are presented in Table 4.

Harrell’s C-index showed modest discrimination for OS and PFS, and higher discrimination for LT. For OS, C was 0.57 with Aza-only, 0.58 with IPSS-R alone, and 0.61 for the full model (Aza, IPSS-R, albumin; 95% CI, 0.57–0.71 by bootstrap). For PFS, C was 0.56 with Aza-only, 0.59 with IPSS-R alone, and 0.66 for the full model (Aza, IPSS-R, marrow blasts; 95% CI, 0.58–0.75). For LT, C was 0.58 with Aza-only, 0.61 with IPSS-R alone, and 0.70 for the full model (Aza, IPSS-R, marrow blasts, age; 95% CI, 0.63–0.82). Overall, adding clinical covariates to Aza or IPSS-R improved discrimination, particularly for LT.

## 4. Discussion

Higher-risk myelodysplastic neoplasms carry a substantial risk of leukemic transformation and early death. In this single-center, real-world cohort restricted to IPSS-R High/Very High disease, azacitidine exposure was consistently associated with improved overall survival and progression-free survival compared with best supportive care alone. The direction of effect was also favorable for leukemic transformation, including when we accounted for death as a competing event. Notably, treatment associations strengthened after multivariable adjustment, suggesting that baseline differences may have attenuated the unadjusted estimates.

To make the treatment effect easier to interpret, we reported both relative measures (hazard ratios) and time-specific differences from Kaplan–Meier curves. At 12 months, estimated OS was 50% with azacitidine vs. 33% without azacytidine; at 24 months, OS was 27% vs. 14%. For PFS, the corresponding 12- and 24-month estimates were 41% versus 28% and 22% vs. 6%, respectively. These absolute separations are modest but clinically meaningful in a population with heavy early event burden. Importantly, crude event proportions were similar between groups, yet time-to-event analyses favored azacitidine. This is not contradictory: event proportions ignore when events occurred and how long patients were followed. In settings with rapid early events and heterogeneous follow-up, time-to-event methods are necessary to quantify the timing of adverse outcomes and to avoid misleading comparisons based solely on proportions.

Overall survival in our azacitidine-exposed group (median 13 months) was shorter than the 24 months reported in AZA-001 [14] and toward the lower end of real-world estimates [16,17,18,19], but the direction of benefit remained consistent. Several factors can explain this difference, which is to be expected when comparing a randomized trial to a real-world retrospective cohort. First, we measured OS from the date of diagnosis, capturing very early post-diagnosis deaths that are often not represented in randomized trials (e.g., in AZA-001, OS was measured from randomization). Second, our cohort had markers of advanced disease and limited reserve, including a median hemoglobin at diagnosis of 7.2 g/dL and a high rate of transfusion dependence (82%). This baseline burden can shorten OS even with active therapy. Third, azacitidine exposure was heterogeneous, with a median treatment duration of 6 months and only half of treated patients receiving at least six cycles. Trial protocols typically deliver more standardized dosing and monitoring and often achieve longer median exposure, which can translate into longer observed survival. Finally, “higher-risk” definitions differ across studies; we restricted inclusion to IPSS-R High and Very High categories, which may enrich more adverse disease biology compared with older IPSS-based definitions [4,26]. This could mean our cohort included patients with more severe disease on average, which would shift OS downward. Despite these differences, our results are consistent with AZA-001 in direction.

Progression-free survival is clinically meaningful in higher-risk MDS, particularly when defined as the earliest of leukemic transformation or death, because it captures the two competing adverse outcomes in this setting. Using this definition, azacitidine exposure was associated with a longer median PFS (11 vs. 7 months) and improved PFS over time. The clinical interpretation is that azacitidine was associated with a delay in either AML evolution or death, whichever occurred first. This endpoint is particularly relevant in older, frail cohorts where competing mortality is common, and it avoids some of the heterogeneity introduced when “progression” is defined using less specific or variably captured clinical criteria [27,28]. Our findings are consistent with randomized evidence such as CALGB 9221, where azacitidine prolonged time to transformation or death (21 vs. 13 months) [23], while the shorter medians in our cohort likely reflect the real-world case mix and treatment exposure heterogeneity.

Leukemic transformation is another central endpoint in higher-risk disease, but it must be interpreted in the context of competing mortality. In many retrospective series, transformation is reported either as an event proportion or using Kaplan–Meier methods that censor deaths, which can be misleading when death occurs early and precludes observation of AML evolution. We therefore reported transformation-free survival and, in parallel, competing-risk cumulative incidence (Aalen–Johansen) to estimate the probability of transformation before death. In our cohort, azacitidine exposure showed a consistent signal toward delayed transformation. Kaplan–Meier LTFS favored azacitidine (median 13 vs. 8 months; *p* = 0.0581), and competing-risk estimates showed a lower cumulative incidence of transformation at both 12 and 24 months in the azacitidine group. These findings are directionally consistent with trial evidence that azacitidine can delay AML evolution [14,23,24] and they highlight the value of competing-risk reporting when transformation is the endpoint of interest.

Our findings should also be interpreted in relation to the Spanish registry study by Bernal et al. [16], which represents one of the largest retrospective real-world comparisons of azacitidine in higher-risk MDS. In that multicenter analysis of 821 patients, 251 received azacitidine and 570 received conventional care treatment, the latter including best supportive care, AML-type chemotherapy, and a small number of other therapies. Median OS was 13.4 months in the azacitidine group versus 12.2 months in the conventional care group (*p* = 0.41), and azacitidine was not independently associated with improved OS in multivariable analysis. Likewise, no significant effect on AML transformation was demonstrated (*p* = 0.42). In contrast, our study found a more favorable association for azacitidine after adjustment. Several design differences may explain this. First, Bernal et al. compared azacitidine with a heterogeneous conventional-care group—best supportive care, but also intensive AML-type chemotherapy and other active therapies, including lenalidomide and cyclosporine—whereas our comparator consisted of an explicit no-azacitidine/best supportive care group. Second, our cohort was restricted to IPSS-R High and Very High risk disease, whereas Bernal et al. used higher-risk disease defined by the original IPSS. Third, our single-center design reduced inter-center variability in supportive care practice and follow-up, although at the cost of a much smaller sample size. Taken together, the apparent discrepancy between Bernal et al. and our findings does not represent a contradiction but rather reflects how cohort composition, comparator arm definition, and analytical strategy each independently shape observed treatment effects in real-world MDS research. Our findings extend those of Bernal et al. by suggesting that, in a more homogeneous and uniformly adverse-risk population receiving BSC as the sole alternative to azacitidine, a measurable survival benefit may be more readily detectable, consistent with the known mechanism of benefit through disease stabilization and delayed deterioration rather than remission induction alone.

Beyond treatment exposure, our analyses emphasize that meaningful prognostic heterogeneity persists within the “higher-risk” label. Even after restricting inclusion to IPSS-R High/Very High categories, IPSS-R risk group remained an independent and dominant driver of outcome across OS, PFS, and LT. Moving from High to Very High risk was associated with a substantially worse hazard of death, PFS events, and transformation in our multivariable models. Clinically, this reinforces that “High” and “Very High” risk patients should not be given the same expectations, which supports improved prognostic refinement (e.g., IPSS-M [6]).

Marrow blast percentage at diagnosis emerged as an independent predictor of leukemic transformation in our purposeful-selection model (HR 1.08 per 1% increase). This pattern is biologically coherent. Blast burden is a disease-proximal variable that reflects proximity to the AML boundary and ongoing clonal evolution, and it therefore plausibly tracks more closely with transformation timing than with all-cause mortality. In contrast, OS and composite PFS are influenced not only by disease biology but also by competing causes of death and baseline physiologic reserve. Our finding that blasts were most strongly linked to transformation is consistent with the way blast percentage functions within established prognostic systems [4], with the clinical view that higher-risk MDS and AML with MDS-related features exist on a continuum [29].

Host factors also mattered in our models. Albumin was retained in the final OS model because its removal meaningfully shifted the treatment effect estimate, suggesting confounding by baseline resilience rather than a purely disease-driven effect. Serum albumin can act as an indirect measure of nutritional reserve, chronic inflammation, comorbidity burden, and frailty, all of which influence supportive care needs, treatment tolerance, and survival. Prior work supports this interpretation: in a large MDS cohort, lower serum albumin at presentation stratified overall survival across IPSS risk groups and remained independently prognostic after adjustment for age and other clinical variables [30]. Age showed a similar role in LT modeling: it was retained because it influenced the treatment estimate, even though it was not independently significant, consistent with the idea that age mainly acts through competing risks and treatment selection. Older patients may die before transformation is observed, and they are less likely to receive intensive or sustained therapy. In the original IPSS-R work, age was a significant additive factor for survival but not for transformation, reinforcing the idea that “patient fitness” and “disease biology” can separate when LT is the endpoint [4].

Treatment delivery in routine practice was heterogeneous, and most azacitidine-treated patients achieved stable disease rather than formal CR/PR at the six-month assessment. This is an important practical point. Responses to azacitidine can be delayed, and clinically meaningful benefit may occur without CR/PR. Practical recommendations derived from trial experience suggest continuing therapy for at least 4–6 cycles in the absence of progression or unacceptable toxicity, with many responses emerging within the first six cycles [31]. In AZA-001, survival benefit was observed even among patients with hematologic improvement without CR/PR, and stable disease was associated with a lower risk of death relative to progressive disease [32]. Our response distribution (predominant stable disease) is therefore compatible with a mechanism of benefit through disease control and delayed deterioration rather than remission alone.

We recorded the number of cycles and treatment duration, but we did not include them as covariates in survival models, because patients must survive long enough to accumulate additional cycles; treating cycle count as a baseline covariate would introduce immortal time bias and can create a false association between higher cycle counts and better survival [33]. The observed heterogeneity in exposure likely attenuates effect sizes compared with trials, while still allowing clinically relevant benefit in routine care. The strengthening of azacitidine associations after adjustment further suggests that baseline differences between treated and untreated patients may have biased unadjusted estimates toward null. We used a purposeful selection strategy that prioritizes control of confounding (including retaining variables that materially shift the treatment estimate) rather than purely *p*-value-driven sparsity.

Several methodological strengths support the interpretability of our findings. The single-center design provides internal consistency in diagnostic work-up, follow-up practice, and supportive care, and it enables a treated-versus-untreated comparison within the same setting. Endpoints were prespecified and clinically anchored, with parallel evaluation of OS, PFS, and LT using standard time-to-event methods, and LT was analyzed with relevant attention to competing mortality by reporting both Kaplan–Meier LTFS and Aalen–Johansen cumulative incidence.

At the same time, several limitations should be emphasized. The study was retrospective, single-center, and non-randomized, which limits causal inference and generalizability and leaves open the possibility of residual confounding and confounding by indication despite multivariable adjustment. The sample size was modest, which may reduce precision of effect estimates and limit subgroup analyses. Molecular profiling was not available in a form that would permit IPSS-M classification, leaving additional biological heterogeneity unaccounted for. Finally, leukemic transformation ascertainment in routine practice depends on the timing of marrow reassessment, which may introduce some imprecision in transformation dating.

Overall, within the constraints of a single-center retrospective cohort, azacitidine exposure was consistently associated with improved survival and a concordant signal for delayed leukemic transformation compared with best supportive care. These findings support azacitidine use in routine care while highlighting the importance of consistent endpoint definitions and competing-risk reporting for transformation outcomes, and the persistent heterogeneity that remains even within IPSS-R higher-risk disease.

## 5. Conclusions

In this single-center, real-world cohort of 72 patients with IPSS-R High/Very High MDS, azacitidine exposure was consistently associated with improved overall survival and progression-free survival compared with best supportive care. Leukemic transformation analyses showed a concordant favorable direction, including when death was treated as a competing event, supporting the idea that azacitidine may delay AML evolution in routine practice. Prognostic heterogeneity persisted within higher-risk disease: IPSS-R category remained a dominant predictor across endpoints, and baseline marrow blast percentage independently predicted transformation risk. Taken together, these data support azacitidine as a valuable disease-modifying option for higher-risk patients while emphasizing the need for multicenter validation, molecularly integrated risk refinement, and consistent competing-risk reporting for leukemic transformation.

These findings should be interpreted cautiously and viewed as real-world observational evidence that supports, but does not prove, a beneficial association of azacitidine with clinical outcomes in higher-risk MDS.

## Figures and Tables

**Table 1:** Baseline characteristics by treatment group (Aza vs. No Aza).

Variables	Overall (Median [IQR])	Aza	No Aza	*p*-Value
Age	71 [65–76]	69.5 [65–74]	73 [66.75–81]	0.089
Sex	F: 32 (44.4%);	F: 19 (43.2%);	F: 13 (46.4%);	0.787
	M: 40 (55.6%)	M: 25 (56.8%)	M: 15 (53.6%)	
Hemoglobin (g/dL)	7.20 [6.50, 8.45]	7.20 [6.70, 8.30]	6.90 [6.20, 9.60]	0.540
Transfusion dependence ^1^	No: 13 (18.1%);	No: 4 (9.1%);	No: 9 (32.1%);	0.013
	Yes: 59 (81.9%)	Yes: 40 (90.9%)	Yes: 19 (67.9%)	
Marrow blasts (%)	11 [6–16]	11 [6–16]	8.5 [6–14.5]	0.400
Albumin (g/dL)	4.20 [3.88–4.40]	4.20 [3.80–4.49]	4.30 [3.90–4.36]	0.787
LDH ^2^ (u/L)	451 [254–653.50]	450.50 [244–655.25]	496 [320.50–618]	0.626
Ferritin (ng/mL)	570 [220.50–916.50]	515 [195–914.50]	680 [278.50–1042]	0.486
IPSS-R ^3^ risk category	HR: 33 (45.8%);	HR: 16 (36.4%);	HR: 17 (60.7%);	0.043
	VHR: 39 (54.2%)	VHR: 28 (63.6%)	VHR: 11 (39.3%)	
Karyotype risk ^4^	VP: 9 (12.5%);	VP: 6 (13.6%);	VP: 3 (10.7%);	0.197
	P: 5 (6.9%);	P: 3 (6.8%);	P: 2 (7.1%);	
	I: 29 (40.3%);	I: 22 (50.0%);	I: 7 (25.0%);	
	G: 27 (37.5%);	G: 12 (27.3%);	G: 15 (53.6%);	
	VG: 2 (2.8%)	VG: 1 (2.3%)	VG: 1 (3.6%)	

^1^ Transfusion dependence defined as four or more units of blood required over a period of 8 weeks. ^2^ Abbreviations: LDH, lactate dehydrogenase. ^3^ Abbreviations: HR, high risk; VHR, very high risk. ^4^ Abbreviations: VP, very poor; P, poor; I, intermediate; G, good; VG, very good.

**Table 2:** OS, PFS, and LT: medians and milestone survival at 12 and 24 months by azacitidine exposure (Aza vs. No Aza).

Endpoint	Group	Median (mo)	S (12 mo)	S (24 mo)	*n*
OS	Aza	13.0	0.50	0.27	44
OS	No Aza	10.0	0.33	0.14	28
PFS	Aza	11.0	0.41	0.22	44
PFS	No Aza	7.0	0.28	0.06	28
LT	Aza	13.0	0.53	0.29	44
LT	No Aza	8.0	0.40	0.11	28

**Table 3:** Treatment effect (Aza vs. No Aza)—unadjusted and adjusted Cox models.

Endpoint	Model	HR (Aza vs. No Aza)	95% CI	*p*-Value	*n*
OS	Unadjusted	0.55	0.32–0.95	0.031	72
OS	Adjusted	0.39	0.22–0.72	0.003	72
PFS	Unadjusted	0.59	0.34–1.01	0.053	72
PFS	Adjusted	0.39	0.20–0.73	0.003	72
LT	Unadjusted	0.57	0.31–1.05	0.071	72
LT	Adjusted	0.30	0.14–0.63	0.001	72

**Table 4:** Independent predictors–multivariable Cox model (purposeful selection).

Endpoint	Variable	HR	CI Lower	CI Upper	*p*-Value
OS	IPSS-R risk category	2.68	1.40	5.129	0.003
OS	Azacitidine treatment	0.48	0.25	0.901	0.023
OS	Albumin	0.76	0.40	1.461	0.417
PFS	Azacitidine treatment	0.34	0.18	0.638	0.001
PFS	IPSS-R risk category	2.61	1.42	4.808	0.002
PFS	Medullary blasts %	1.04	0.98	1.109	0.173
LT	Azacitidine treatment	0.23	0.10	0.488	0.000
LT	IPSS-R risk category	3.46	1.67	7.161	0.001
LT	Medullary blasts %	1.08	1.01	1.164	0.026
LT	Age	0.99	0.96	1.011	0.298

## Data Availability

The original contributions presented in this study are included in the article. Further inquiries can be directed to the corresponding author.

## Abbreviations

The following abbreviations are used in this manuscript:

MDS	Myelodysplastic Syndromes/Neoplasms
AML	Acute Myeloid Leukemia
IPSS	International Prognostic Scoring System
IPSS-R	Revised International Prognostic Scoring System
WPSS	WHO Prognostic Scoring System
IPSS-M	Molecular International Prognostic Scoring System
ESAs	Erythroid Stimulating Agents
HMAs	Hypomethylating Agents
OS	Overall Survival
PFS	Progression-Free Survival
LT	Leukemic Transformation
BSC	Best Supportive Care
CI	Confidence Interval
HR	Hazard Ratio
ANC	Absolute Neutrophile Count
IQR	Interquartile Range
WHO	World Health Organization
CR	Complete Response
PR	Partial Response
SD	Stable Disease
PD	Progressive Disease
